# Sleep Deprivation and Oxidative Stress in Animal Models: A Systematic Review

**DOI:** 10.1155/2015/234952

**Published:** 2015-04-06

**Authors:** Gabriel Villafuerte, Adán Miguel-Puga, Eric Murillo Rodríguez, Sergio Machado, Elias Manjarrez, Oscar Arias-Carrión

**Affiliations:** ^1^Unidad de Trastornos del Movimiento y Sueño (TMS), Hospital General Dr. Manuel Gea González/IFC-UNAM, 14080 Ciudad de México, DF, Mexico; ^2^Laboratorio de Neurociencias Moleculares e Integrativas, Escuela de Medicina, División Ciencias de la Salud, Universidad Anáhuac Mayab, 97310 Mérida, YUC, Mexico; ^3^Laboratorio de Pánico y Respiración, Instituto de Psiquiatría, Universidade Federal de Rio de Janeiro (IPUB/UFRJ), 22410-003 Rio de Janeiro, RJ, Brazil; ^4^Instituto de Fisiología, Benemérita Universidad Autónoma de Puebla, 72570 Puebla, PUE, Mexico

## Abstract

Because the function and mechanisms of sleep are partially clear, here we applied a meta-analysis to address the issue whether sleep function includes antioxidative properties in mice and rats. Given the expansion of the knowledge in the sleep field, it is indeed ambitious to describe all mammals, or other animals, in which sleep shows an antioxidant function. However, in this paper we reviewed the current understanding from basic studies in two species to drive the hypothesis that sleep is a dynamic-resting state with antioxidative properties. We performed a systematic review of articles cited in Medline, Scopus, and Web of Science until March 2015 using the following search terms: *Sleep or sleep deprivation and oxidative stress, lipid peroxidation, glutathione, nitric oxide, catalase or superoxide dismutase*. We found a total of 266 studies. After inclusion and exclusion criteria, 44 articles were included, which are presented and discussed in this study. The complex relationship between sleep duration and oxidative stress is discussed. Further studies should consider molecular and genetic approaches to determine whether disrupted sleep promotes oxidative stress.

## 1. Introduction

Sleep is defined as an active state characterized by reduced alertness and responsiveness that is rapidly reversible [1–3]. In spite of not having certainty about its true purpose, there are several indications which infer that sleep is indeed functionally important: (1) sleep is ubiquitous among mammals, birds, and reptiles; (2) sleep has persisted in evolution even though it is apparently maladaptive with respect to other functions; (3) accommodations are made to permit sleep in different environments and life styles; (4) sleep is homeostatically regulated (sleep deprivation is followed by sleep compensation); (5) serious physiological changes result from prolonged sleep deprivation of animals [4, 5]. It has been reported that sleep deprivation represents a serious physiological issue since it causes a state of high caloric ingestion without weight gaining [5–9], reduction in anabolic hormones [10], opportunistic infections, and eventually death [11], among other physiological challenges. Different approaches of prolonged wakefulness have been employed to study the physiological impact of sleep deprivation on neurobiological functions.

Sleep deprivation could be responsible for recruiting neurobiological mechanism related with stress as well as oxidative processes. However, it is highly unlikely that only the adrenergic activation produced by stress mediates the physiological effects described in sleep deprived rats. This observation is based on the physiological disturbances observed after sleep deprivation which are reversed once animals are allowed to sleep* ad libitum* [12]. Therefore, it has been suggested that sleep deprivation* per se* might be the responsible cause of the homeostatic disturbances observed in diverse conditions of prolonged wakefulness [13]. Here, we hypothesized that sleep may have several neurophysiological functions; that is, when impaired, sleep deprivation determines the subject to activate neurophysiological mechanisms to induce significant change in several systems. The hypothetical functions remain unknown until date; however, many different theories have been proposed including the hypothesis that sleep may trigger antioxidative mechanisms [14, 15].

Regarding the oxidative processes, it is known that oxygen is an essential molecule in all aerobic organisms. It is involved in a variety of physiological reactions, such as those produced in the electron transport chain, hydroxylation, and oxygenation. Approximately 1% of the oxygen used in cells is transformed into free radicals (chemical species with an unpaired and highly reactive electron), usually called reactive oxygen species (ROS). The ROS, such as the superoxide anion radical (O_2_^•−^), the hydroxyl radicals (^•^OH), and the nonradical hydrogen peroxide (H_2_O_2_), are all by-products formed as part of the normal aerobic metabolism of the mitochondria and peroxisomes [16]. Antioxidants are a structural heterogeneous group that share the ability to scavenge free radicals and are the first defense against the potential damage of ROS [17]. One of the most significant endogenous antioxidants is the tripeptide glutathione (GSH), which is oxidized by different radicals into glutathione disulfide (GSSG) and confers protection mainly in highly metabolic tissues. Catalase (CAT), superoxide dismutase (SOD), and glutathione peroxidase (GPx) are the most studied enzymes in charge of transforming free radicals into more stable chemical forms and along with glutathione and lipid peroxidation constitute the main antioxidant defenses of animals [18].

Oxidative stress occurs once the burden of prooxidants such as ROS exceeds the antioxidant systems of the body. According to the literature, it can be generated by an enhancement in the concentration of prooxidants, an impaired antioxidant defense, or combination of both conditions [19]. This imbalance leads to a potential damage to lipids, proteins, and DNA as described in aged conditions such as carcinogenesis and neurodegenerative diseases [20].

Wakefulness involves high neuronal metabolism to maintain neuronal electrical potentials, which requires a great amount of oxygen, resulting in a significant production of oxidants. Thus, sleep represents a state with an increased antioxidant activity which promotes a brain protection against free radicals via a diminution in oxidant production [15]. The ROS and other oxidative stress markers could be accumulated in the brain tissue during wakefulness, and after reaching out a threshold, they could behave as sleep promoters [21].

Although sleep has been described in most (if not all) mammals, still there are not any methods to predict the duration of a sleep episode. Several approaches have drawn putative mechanism regarding the sleep duration. It has been suggested that the relationship between weight and sleep could play a key role. For instance, less body weight represents a higher metabolic rate as well as sleep, and probably this relationship could induce higher oxidative stress activity [4]. It is a provocative theory of sleep function since it links the ubiquitous sleep activity with ROS [15].

Because sleep includes different sleep stages with specific neuronal activity, further complexity is included in its physiological mechanisms. For example, paradoxical sleep (PS, also called rapid eye movement (REM) sleep) is associated with high neuronal metabolic activity and loss of muscular tone, whereas slow-wave sleep (SWS) is characterized by low neuronal metabolic activity [22]. Moreover, sleep duration and sleep stages display different patterns among the studied mammals so far, suggesting a complex function probably dependent on the ecological niche in which each animal has evolved. Furthermore, it has been described that during sleep there are different brain regions exhibiting specific electrical activity, suggesting that the regional changes in the metabolic activity of the brain are different. Therefore, behavioural changes observed in sleep deprived animals could be explained by regional oxidative stress in the brain [23].

Even when research on sleep function has been carried on since 1950s, the methodological issues that come along with the difficulties to dissect such a complex phenomenon as sleep made conclusions mere speculations; bias came predominantly from the difficulty to achieve stimulus that could keep an experimental subject awake while not causing other deleterious effects on itself or in a control (and also allowing the control subject to sleep). The disk over water (DOW) deprivation method designed in 1983 was the first method to achieve the characteristics described before [8, 24] and nowadays, along with the multiple small platforms (MsP) method, is the most used mechanical method to study sleep deprivation.

Taking together these data, in the present report we addressed evidence that supports the hypothesis that sleep is a dynamic-resting state with antioxidative properties. Moreover, differences between studied species in the oxidative stress after prolonged waking or brain regions affected by oxidative stress after sleep deprivation are discussed. Since we want to study antioxidant properties of sleep, mechanical methods of sleep deprivation are the ones selected in order to avoid bias that could be given by pharmaceutical deprivation (adverse effects, difference between drugs metabolism and elimination between strain and species, inhibition/stimulation of antioxidant signaling, etc.). In order to accomplish the aim of the present paper we also contemplated in our revision evidence that suggests that oxidative stress may be the inductor of sleep and thus confirmed its antioxidant properties.

## 2. Methods

### 2.1. Search Strategy

A systematic review of the literature following the PRISMA model (preferred reporting items for systematic reviews and meta-analysis) was carried out. The literature included in the analyses comprised articles from the Medline, Scopus, and Web of Science electronic databases updated until March, 2015. The MeSH terms (medical subject headings) used for the search were “sleep” or “sleep deprivation” and “oxidative stress,” “lipid peroxidation,” “glutathione,” “nitric oxide,” “catalase,” or “superoxide dismutase.” The precise search algorithm used on the Medline database is as follows: ((“sleep” [MeSH Terms]) OR (“sleep deprivation” [MeSH Terms])) AND ((“oxidative stress” [MeSH Terms]) OR (“lipid peroxidation” [MeSH Terms]) OR (“glutathione” [MeSH Terms]) OR (“nitric oxide” [MeSH Terms]) OR (“catalase” [MeSH Terms]) OR (“superoxide dismutase” [MeSH Terms]) OR “superoxide dismutase” [All Fields]) NOT ((“neurodegenerative diseases” [MeSH Terms]) OR (“sleep apnea syndromes” [MeSH Terms]) OR (“pregnancy” [MeSH Terms]) OR (“obesity” [MeSH Terms]) OR (“lung diseases” [MeSH Terms]) OR (“stroke” [MeSH Terms]) OR (“heart diseases” [MeSH Terms]) OR (“pulmonary disease, chronic obstructive” [MeSH Terms])) NOT (“review” [Publication Type] OR “review literature as topic” [MeSH Terms] OR “review” [All Fields]) AND “animals” [MeSH Terms:noexp]. Articles included in the analysis focused on sleep deprivation or sleep promotion, as well as relationship among sleep and ROS, or antioxidative measurements. The articles retrieved were evaluated by two independent reviewers; furthermore, topic filters were used (analysis of the title, summary, and critical analysis of the full-text article). The search was limited to original articles referred to as experimental evidence reported with animals, published in English language with no year restriction. Further analysis of the references of each article was also performed, aimed at finding articles that could have not been included by the algorithm. Due to repetitive data already published in specific reviews about the topics, data regarding the role of nitric oxide and the sleep-wake cycle were excluded [25, 26].

### 2.2. Analysis of the Data

**Figure 1:**
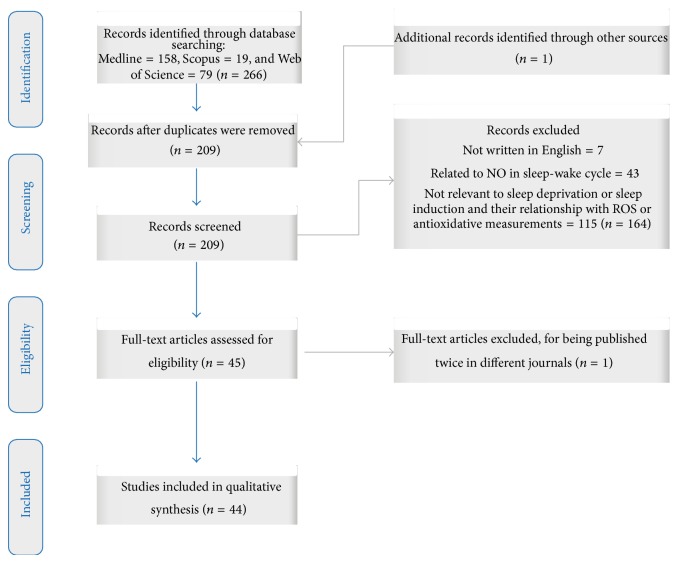
Flowchart for articles selection.

A total of 266 papers were retrieved from the databases and 1 additional article was retrieved after the extended search of the references from each paper. Cross-referencing was carried out to eliminate duplicated references and a total of 57 references were excluded. From the 209 remaining papers, 114 were excluded because of nonfocus on sleep deprivation or sleep induction and their relationship with ROS or antioxidative measurement. Next, 43 papers were excluded due to nitric oxide and sleep-wake cycle topics. Next, 7 published papers were excluded because of language edition, and finally 1 article was excluded because of being published in two different journals. The remaining 44 studies [27–70] were included in the final qualitative analysis (see Figure 1).

## 3. Results

It was found that 32 papers used a sleep deprivation protocol for the measurement of oxidative stress in different brain regions. There are 2 articles describing sleep deprivation protocols according to the sleep stage to be deprived [71, 72]; however, methods to accomplish particular sleep stage deprivation vary between articles dependent upon objectives established. According to our analysis, the REM sleep deprivation protocol (or paradoxical sleep deprivation [PSD] protocol) was the method most consistently employed [27, 28, 30–35, 37, 40–52, 56–58]. The PSD protocols are summarized in Table 1.

On the other hand, to block SWS, it was found that the most reported protocol is the total sleep deprivation (TSD) procedure [29, 36, 38, 39, 53–55]. The TSD data is summarized in Table 2. Because GSH, GSSG, GSH/GSSG ratio, GPx, CAT, SOD, nitrites, NO, and lipid peroxidation are the most practical procedures to measure oxidative stress, these neurobiological markers were included in the analysis of the literature. Based on our data, different areas of the brain showing disturbances compared to control in the sleep deprived animals were identified, including reports showing subjects with different times of sleep deprivation. The results are summarized in Tables 3, 4, and 5.

Since there are differences in sleep patterns (even within strains) [73] as well as in the antioxidant metabolism between rats and mice, different analyses for PSD were considered. For example, 12 articles of mice under PSD procedures [27, 40, 41, 43–47, 49–52] and 13 papers of rats after PSD methods [28, 30–35, 37, 42, 48, 56–58] were analyzed. However, TSD protocols were described exclusively in rats [29, 36, 38, 39, 53–55].

Moreover, 13 papers were focused on the antioxidant measure on the whole brain [35, 40–48, 50, 51, 58], 2 articles described the issue on the striatum [27, 42], 17 reports included the hippocampus [27–29, 31–38, 42, 49, 52, 54, 55, 57], 12 papers reported the antioxidants in the cortex [27, 28, 30, 34, 36–38, 42, 53, 55, 57, 58], 1 article was aimed at the amygdala [34], 4 articles addressed the issue on hypothalamus [42, 55–57], 2 reported papers described it at the thalamus [42, 57], 4 reports mentioned the brainstem [36, 38, 42, 55], 4 articles reported the antioxidant activity in the cerebellum [36, 38, 55, 57], 1 paper demonstrated the link in the pons [57], and finally 1 report published the association among antioxidant levels and forebrain [38].

In our analysis of the literature, we found that the time of sleep deprivation ranged from 6 h to 14 days, depending upon research goals. In PSD mice, the procedure was reported in 10 studies as a standard of 72 h [40, 41, 43–47, 50, 52]. We also found that 2 studies reported a PSD of 48 h [27, 51] and 1 study used a sleep deprivation procedure of 24 h [49]. On the other hand, the analysis of PSD in rats showed that this experimental manipulation had a duration of 96 h in 5 studies [42, 48, 56–58]. Three reports described the use of PSD of 24 h [28, 30, 34], whereas 2 papers reported the use of TSD of a time of 72 h [28, 37]. Moreover, 4 studies used a noncontinuous deprivation time, whereas 3 reports showed sleep deprivation time of 8 h daily during 6 weeks [31–33]. A single paper was found describing the use of PSD of 18 h daily during 21 days [35]. Our meta-analysis showed that, in those reports using TSD procedures in rats, the common feature is prolonged waking of 6 h [30, 36, 38, 39]. Several reports showed different time of sleep deprivation, including 24 h [29], 5 days [54], 8 hrs–14 days [53], and 5–11 days [55].

Finally, the analysis of the literature showed that 8 papers reported the sleep deprivation protocol for the measurement of oxidative stress out of the brain areas [53, 59–64, 70]. The results are summarized in Table 6. In these reports, the common region was also the liver [53, 61, 63], whereas different papers reported biological samples such as skin, lung, heart, spleen, skeletal muscle, plasma, and nodose ganglion [53, 59, 60, 62–64]. It was found out that PSD protocols were used in 3 studies [59, 60, 64], whereas TSD procedures were reported in 4 papers [53, 61–63]. The antioxidants studied were GSH, GSSG/GSH ratio, GPx, CAT, SOD, and lipid peroxidation. The time of sleep deprivation ranged from 72 h to 14 days. Importantly, 1 study [70] was not included in Table 6 since it was not focused on any of the antioxidant markers; however, it was focused on genome expression of several antioxidant molecules and it was considered for the present revision [59]. Finally, our analysis showed that 5 papers studied the induction of sleep mediated by oxidative stress by other ways apart from NO [65–69].

All results showed in Tables 3, 4, 5, and 6 are from measures of the antioxidants in sleep deprivation alone. Even when the focus of any of the particular articles was to compare effects of different conditions and/or drugs, results taken were exclusively those comparing sleep deprivation alone and controls.

## 4. Discussion

### 4.1. Neurobiological Differences among Two Species in Oxidative Stress in Brain after Sleep Deprivation

We found 32 papers related to sleep deprivation and measurements of oxidative stress in the brain. Four different species were used systematically in the studies assessed: Wistar rats (16), Sprague Dawley rats (5), Swiss mice (3), and Laca mice (9).

Sprague Dawley rats were used in 2 studies with TSD using the DOW protocol (see Table 2) during 5–11 days and GPx, SOD, and NO [54, 55]. Significantly, one of these reports found a diminution in SOD in the hippocampus and brainstem [55], whereas Gopalakrishna and colleagues showed no significant differences in the concentration of lipid peroxidation and SOD after sleep deprivation in Wistar rats [53]. Although the results comparing Sprague and Wistar rats might be related to strain differences, we hypothesize that the lack of significant difference of SOD activity in Wistar rats may be attributable to different areas of the brain studied (hippocampus* versus* cerebral cortex).

Sprague Dawley rats have been studied using protocols of TSD. It was found that one study analyzed the oxidative stress after short term of sleep deprivation [38], whereas other reports used short term sleep deprivation under sustained hypoxia [36]. In both articles, an increase in antioxidative activity was found, suggesting that short term sleep deprivation involves compensating mechanisms to protect the brain from oxidative stress. Similar result was observed using Wistar rats after TSD protocol; however, in this report animals were under TSD during 24 h by handling manipulation leading to a diminution in lipid peroxidation in the hippocampus [29]. Taken together, these results suggest that antioxidative mechanisms are activated after TSD in Sprague Dawley as well as in Wistar rats [36].

Further evidence shows that one study with a PSD protocol used Sprague Dawley rats [28], whereas the rest of the papers used Wistar animals. Briefly, one of the PSD articles employed Wistar rats under the same sleep deprivation protocol (MSP with MLP), with a similar time for sleep deprivation (72 h), similar collected brain areas (hippocampus and cortex), and the same oxidative stress markers [37]. Comparative studies showed that Sprague Dawley and Wistar rats displayed a significant increase in lipid peroxidation in hippocampus and cortex, suggesting no significant differences in oxidative processes between each strain. Based on our meta-analysis, it is not possible to draw a solid conclusion that no difference between Sprague Dawley and Wistar rats is present after sleep deprivation and antioxidative processes; however, the current knowledge suggests similar patterns of oxidative stress between these two strains.

On the other hand, methodological and theoretical aspects limit the comparison among Laca and Swiss mice after sleep deprivation and biological antioxidative markers because of the sleep deprivation method (see Tables 1 and 3).

In order to find out significant differences between rats and mice, in oxidative processes after sleep deprivation, we focused on those studies using similar sleep deprivation protocols in both species. Since no reports of TSD in mice were found, the analysis of sleep deprivation and antioxidative processes was limited. However, 3 studies of PSD were described in Swiss mice using the MSP method. The principal findings of such studies were an increase in hippocampal lipid peroxidation [27, 52] and a decrease in GSH concentration [52] compared to controls [49] after 48 h of sleep deprivation. Remarkably, these findings were not observed after 24 h of sleep deprivation. Further similar results were found in rats. For instance, PSD with the MSP method during 24 h increased the lipid peroxidation in the hippocampus [34]; however, a 72 h of sleep deprivation with the same experimental protocol produced a significant enhancement of the lipid peroxidation in the hippocampus [28, 37].

In reports using mice it was described that prefrontal cortex shows lipid peroxidation after more than 48 h of PSD [27]. Similar data has been found in rats [28, 37]. Despite this remarkable result, differences should be highlighted. For example, after 24 h of sleep deprivation, a change in lipid peroxidation rate is observed in rats, while mice showed no change in this molecular marker. Despite the fact that the papers do not show a mechanism of action, it can be hypothesized that differences among species could be related with strain-dependent mechanisms. Alternative explanations about the effects of sleep deprivation would represent a biological challenge greater for mice than for rats.

The increase in lipid peroxidation rate could be the result of the amount of sleep rather than the consequence of sleep deprivation. Thus, it would be expected that if the sleep function is related to eliminate ROS, the animal which displays higher sleeping time periods should display higher amount of ROS even after sleep deprivation. We do believe that future studies regarding the description of effects of sleep deprivation and endogenous molecular markers should consider these issues.

### 4.2. Is There a Differential Role in the Antioxidant Properties of PS and SWS?

As mentioned above, we found a total of 25 studies referring to PSD and 7 reports using TSD. The original hypothesis about the antioxidant properties of sleep referred that SWS would accomplishes for the most antioxidative part of sleep. This assumption would be confirmed once SWS is associated with low neuronal metabolic activity and therefore with less oxygen consumption.

Paradoxical sleep features a neuronal activity similar to the alertness; thus, it would be expected that the oxidative stress generated during REM sleep should display similar rates of oxidative index compared to wakefulness. However, the experimental data extracted from PSD points out to a different perspective: paradoxical sleep plays a significant role as antioxidative element and whenever prevented, oxidative stress increases. From the 12 mice studies in which PSD was generated, 11 papers found an increase in oxidative stress parameters. One paper was found that did not report significant differences in mice in PSD for only 24 h. Further studies are needed to describe if paradoxical sleep in mice shows antioxidant properties.

A remarkable result in our analysis includes that both PSD protocols used (MSP and GOW methods) are reliable. Although both protocols ensure an objective REM sleep deprivation, it also diminishes the amount of SWS [31]. Thus, a question that remains is the following: was the oxidative stress measured generated either by the small amount of SWS deprivation or by the PSD? Unfortunately, we could not find any study with a deprivation protocol that exclusively abolished SWS; therefore, there is not a clear difference between the antioxidant properties of SWS and PS in mice. Another issue that should be highlighted is that most of the studies which referred to PSD in mice were conducted by the same group of researchers (9 out of 12 studies) and some articles share controls [47]. Importantly, the purpose of these papers was to explore the effects of specific drugs and the oxidative stress generated after SD. Under their experimental conditions, control groups were sleep deprived animals with no pharmacological application.

Concerning the sleep deprivation studies that used rats, 11 out of 13 reports showed significant differences in one brain region. Moreover, from these studies, one paper reported a decrease in oxidative stress (decreased lipid peroxidation), whereas 10 papers showed an increase in oxidative stress. Based on this evidence we suggest that the classical platform (CP) method is reliable (see Table 1) for PSD with at least 96 h of sleep deprivation. Oxidative stress was observed in hypothalamus [56, 57] and hippocampus [42]. However, the CP method has methodological issues that predispose the animals to suffer from isolation and immobilization stress, conditions that generate oxidative stress [74, 75]. The MSP with MLP method was the PSD protocol with the proper fashion to control possible environmental variables that generate stress. Current evidence suggests that this protocol generates oxidative stress, with special focus on cortex and hippocampus [28, 34, 37]. Four studies reported a “physiological” approach to generate PSD: instead of prolonged waking, authors used specific time points of the day for sleep deprivation. This experimental approach offers the advantage to reduce the amount of stress intrinsic to the sleep deprivation and represents a reliable model for human sleep deprivation. This modified sleep deprivation protocol also showed an increase of hippocampal lipid peroxidation [31–33, 35].

According to our analysis, the first published article that measured oxidative stress after PSD did not find changes in oxidative stress. However, this result may be caused by two factors: (1) assessment of the oxidative stress in a homogenized sample of the whole brain and not by specific regions (using the whole brain could mask changes in oxidative stress in particular regions of the brain) and (2) use of the CP method that, as previously mentioned, shows methodological issues [58]. The other article that could not find differences in the oxidative stress used a Wistar Hanover rat model, which is different to all other strains; therefore, these results should be regarded aside [30]. The experimental evidence found suggests that rat's paradoxical sleep also has an antioxidant role; however, it struggles with the same issues mentioned in mice: PDS deprivation is accompanied by some degree of SWS deprivation and therefore the bias continues.

TSD has the advantage to show more significant changes in the oxidative stress elements but it lacks efficiency in dissecting the function of each sleep phase. In human, sleep deprivation does not occur in a selective manner. Therefore, TSD is a remarkable model to assess the behavioral and biochemical changes during sleep deprivation conditions. Despite the positive points mentioned before, the experimental evidence regarding TSD and oxidative stress is more complex. The handling method for TSD during 6–24 h shows a reduction in hippocampal lipid peroxidation [29, 36] and an increase of cortical, cerebellar, and brainstem GSH [36, 38]. This discrepancy may be caused by an overcompensation mechanism activated by a short sleep deprivation period which likely occurs at the first 24 h of sleep deprivation. Based on our meta-analysis, we did not find studies using the handling method for TSD during more than 24 h. Basically, if TSD is aimed at being prolonged for more than 24 h, the disc over water (DOW) method is usually used (see Table 1); the reason for this may be that a continuous manual stimulation in a chronic fashion may produce stress by itself and therefore be cofounder. The DOW method was included in 3 papers [53–55] and the results are contradictory. On the one hand, a diminished activity of SOD in the hippocampus was reported [55], whereas on the other hand no difference in brain region was described [53].

The above-mentioned studies suggest that the paradoxical sleep deprivation displays a role in the oxidative function of sleep, whereas the SWS role in oxidative function could not be evaluated, likely due to the lack of an effective method for SWS deprivation. However, the controversial evidence of the TSD experiments suggests that the role of SWS is not as important as assumed previously.

### 4.3. Which Brain Regions Are Preferentially Affected by Oxidative Stress after Sleep Deprivation?

After the qualitative analysis of the 32 sleep deprivation studies, we can conclude that, at least, the rat brain is not uniformly affected by sleep deprivation. Studies using a whole homogenized brain showed changes in oxidative stress in mice [43, 45] but not in rats [58].

Using the whole brain excludes the possibility to describe the specific part of the CNS that is affected by oxidative stress. Likely mice show higher rates of generalized oxidative stress response to PSD by grid over water (GOW) method, but it was difficult to compare with rats results because our search analysis did not retrieve any rat study in which the GOW protocol was used. Until more evidence is available, we cannot discard the possibility that the generalized increase in the oxidative stress parameters of the mice brain is intrinsic to the GOW protocol.

In rat studies the whole brain analysis did not show significant changes in oxidative stress after PSD with the CP protocol [58]. However, a paper that analysed several brain areas found an increase in lipid peroxidation at the hippocampus, thalamus, and hypothalamus but a decrease in lipid peroxidation at the cerebral cortex. Thus, homogenates of brain show no difference [42].

According to our analysis, it was found that hypothalamus [42, 56, 57], hippocampus, and thalamus [42] in the rat exhibited an elevated oxidative stress after PSD in a CP. Other studies show that the hippocampus, amygdala, and cortex are also affected by PSD [28, 34, 37]. Regarding the handling protocol for TSD, it was found that the hippocampus displayed antioxidative enhancement activity but not the cerebral cortex [29, 36, 38].

### 4.4. Antioxidative Role of Sleep Is Observed in Brain and Beyond

Sleep is generally conceived as a function of CNS. Moreover, sleep deprivation studies have demonstrated that sleep deficits also affect other physiological systems [10, 11]. It has been proposed that oxidative stress in other organs could mediate the sleep deprivation syndrome previously described. However, the evidence from our review is not clear. Current evidence shows that several body organs have also been studied after sleep deprivation: skin, lung, heart, spleen, skeletal muscle, plasma, nodose ganglion, and liver [53, 59–64, 70]. Furthermore, differences in oxidative stress after prolonged waking were also found in liver, spleen, nodose ganglion, heart, and plasma [59, 61–64].

The DOW method for 5 and 10 days showed a significant decrease liver's GSH and a decrease in CAT activity [63]. Moreover, there was significant decrease in liver CAT activity from day 5 to day 10. Contradictory results regarding liver lipid peroxidation show an increase in lipid peroxidation in one study [61], whereas other reports show no difference [53]. Liver behaves as the principal detoxifier by supporting the body to get rid of many toxic substances, in which ROS are included. The changes previously mentioned should be explained by 3 different theories: (1) the ROS associated with wake and increased physical activity are accumulated due to the DOW method and the intrinsic antioxidative defenses of liver could have been overwhelmed by the increased concentration of ROS; (2) sleep directly promotes an increase in the antioxidative systems in liver and when sleep deprivation occurs, the enhancement is prevented and the liver is incapable of coping with the normal ROS concentration; (3) a mixture of the previous two statements is observed. Our analysis is limited since no further evidence was found to support the hypothetical mechanism mentioned lines above.

One study assessed the oxidative stress in heart and lung after TSD by the DOW method and found an increase in the heart's GPx. The lung did not show any change in oxidative stress parameters [63]. The increase in GPx could represent a compensatory mechanism to the increased oxidative stress. The heart is an organ with high metabolic activity that has decreased antioxidant defenses [76]. Sleep benefits the brain and the heart by decreasing metabolic activity and reducing burden of oxidative stress. Sleep diminishes the energy consumption of cardiac muscles; thus, the generation of a dynamic-resting state with lower metabolic activity ultimately decreases the metabolic demands of the heart. The DOW method can generate great amounts of stress from sleep deprivation that could increase the oxidative stress of the heart. There is no solid evidence to explain why the GPx activity is increased and if this enhancement is caused by a compensatory mechanism linked with an increase in ROS activity.

Although the lungs did not show any difference in the oxidative patterns after TSD by the DOW method in rats, a study that used the handling method for TSD in mice found that sleep regulated the genes responsible for synthesizing thioredoxin and glutathione S-transferase, both proteins related to antioxidative defenses [70]. This finding suggests that sleep changes the metabolic activity, as well as activating the genetic expression of diverse antioxidative defenses. Analysis of genetic expression paired with the measure of their specific protein should be performed in diverse organs as well as in the brain to clarify the role of sleep in genetic expression of antioxidative defenses.

Finally, we found several reports assessing other systems such as blood [64], skeletal muscle [53], nodose ganglion [62], skin [60], and spleen [59]; however, the antioxidant defenses displayed differences between studies and a comparison among these studies is limited. Further experiments aimed to study the role of antioxidative stress, sleep deprivation, and physiological systems are required.

### 4.5. Does Oxidative Stress Induce Sleep?

During the twentieth century the hypnotoxin theory of sleep, proposed by Pieron, provided a reasonable explanation regarding the origin of sleep. This theory stated that a certain “toxin” would accumulate during waking and after a certain threshold it could induce sleep. The scientific evidence that supported this theory was drawn by experiments with dogs. After sleep deprivation, cerebrospinal fluid was collected and injected into nondeprived dogs. It was found that recipient animals displayed behavioural signs of sleep [77]. This theory has been strengthened by increasing evidence of the existence of several endogenous molecules that accumulate during waking. Thus, it is likely that, during sleep deprivation, an endogenous compound would be accumulating to reach out a point to trigger antioxidative mechanism. In this regard, 5 studies referring to the ability of oxidative stress to induce sleep were found. The identification of the nature of sleep-promoting substance of sleep deprived rats included oxidized glutathione (GSSG), a neurobiological marker of oxidative stress [69]. GSSG is catalyzed by the GPx enzyme using as substrate reduced GSH. This biochemical reaction is carried out to reduce unstable molecules as free radicals [69]. Later, the role of GSSG in sleep induction of the rat was studied by applying intracerebroventricular GSSG. The pharmacological study showed an increase in SWS as well as PS. Despite the fact that a significant enhancement was observed in both sleep stages, the increase in SWS sleep was caused by the enhancement in the duration of SWS episodes, whereas the enhancement in PS time was due to an increase in the number of bouts of PS. Taken together, the results suggest that GSSG differentially modulates sleep stages [68]. Although GSSG behaves as a sleep-inducing molecule, after intravenous administration no sleep effects were found [67].

An increase in the levels in cerebrospinal fluid of GSSG would not be expected as an initial result in oxidative stress generated in control conditions. It is assumed that an increase in oxidative stress in brain would be observed as the first response to ROS generation. Due to the complex relationships of GSSG with many different enzymes and molecules it is difficult to assure that this molecule is a sleep-inducing factor.

Finally, in our revision we found evidence that compounds related to oxidative stress also behave as sleep-promoting molecules. For example, an organic hydroperoxide, *t*-butyl-hydroperoxide (TBHP), is a substance known to promote oxidative stress. It was employed to study the mechanisms of oxidative stress in diverse systems and in a nontoxic dose it promotes sleep in rats [66]. The pharmacological properties of TBHP to promote sleep are similar to GSSG: after administration, TBHP increases the number of PS episodes and increases the duration of SWS episodes. At higher doses, TBHP decreases the duration of sleep and induces neuronal damage. It has been proposed that the sleep-promoting effect of TBHP involves the activity of GSSG [66].

Cadmium chloride is a substance known to increase GSSG and decrease GSH in the brain. It has been demonstrated that contaminated water with this heavy metal given to rats promotes SWS. The GSSG/GSH ratio in rats drinking contaminated water was higher, but not statistically different compared with control animals. However, GSSG was found to increase both PS and SWS. It can be concluded that cadmium chloride could enhance SWS by unknown mechanisms [65].

## 5. Conclusion

Current experimental evidence suggests that sleep deprivation promotes oxidative stress. Furthermore, most of this experimental evidence was obtained from different animal species, mainly rats and mice, using diverse sleep deprivation methods. Differences in the sleep patterns between species and even between strains make it difficult to have a definite conclusion; however, our revision could show that PSD have an important antioxidant function, contrary to the expected results based upon its high neuronal metabolic activity. Aimed at comparing strains and methodological approaches, here we show the evidence regarding the effects of sleep deprivation and the production of antioxidative markers as protective elements. Further studies are needed to address the issues of unknown mechanism of action of sleep deprivation and oxidative stress; SWS function relies without a clear function, since it is difficult to be completely and uniquely abolished. Design of new pharmaceutics or mechanical methods to selectively abolish SWS may give the missing pieces to fulfill the gap in the function of sleep and oxidative stress.

## Figures and Tables

**Table 1:** Paradoxical sleep deprivation protocols.

Sleep deprivation method	Description	Controls	Advantages	Disadvantages
Multiple small platforms (MSP)	Multiple small platforms (3–5 cm) placed in a tank (40 × 30 cm) filled with water to within 1–4 cm of the upper surface of the platforms and spaced 7 cm. Water and food *ad libitum*. The loss of muscle tone results in animals touching the water and awakening	Home caged controls, may use MLP (10–16 cm) controls	Abolishes REM sleep. Eliminates immobilization and isolation stress. If MLP controls are used, then environmental confounds (stress and anxiety) can be controlled	May also decrease slow-wave sleep. Can be affected by environmental confounds (stress and anxiety)

Classical platform (CP)	The animals were individually placed on a platform of 4.5–10 cm diameter in individual containers filled with water up to 1 cm below the platform surface. Water and food *ad libitum*. The loss of muscle tone results in animals falling off the platform and wakening	Home caged controls. WP (13-14 cm) controls	Abolishes REM sleep	May also decrease slow-wave sleep. Isolation and immobilization stress may be present environmental confounds (stress and anxiety)

Grid over water (GOW)	The animals were placed on a grid floor (29 × 15 × 7 cm) inside the plastic cage filled with water to 1 cm below the grid surface. The stainless steel rods of the grid (3 mm wide) were set 2 cm apart from each other. Water and food *ad libitum*. The loss of muscle tone results in animals touching the water and awakening	Grids placed over saw dust controls and home caged controls	Abolishes REM sleep. Eliminates immobilization and isolation stress. Environmental confounds (stress and anxiety) controlled	May also decrease slow-wave sleep

Classical platform: CP, grid over water: GOW, multiple small platforms: MSP, multiple large platforms: MLP, and wide platform: WP.

**Table 2:** Total sleep deprivation protocols.

Sleep deprivation method	Description	Controls	Advantages	Disadvantages
Handling	Rats were kept awake by gently touching their tails or whiskers, brushing their fur, shaking their cages, introducing objects unto their chambers, or disturbing their chamber bedding to prevent them from falling asleep	Home caged controls. EEG and EMG may be used to detect microsleep monitoring. May use handling or home caged controls	Achieves total sleep deprivation with a low stressful environment in an acute mode.	Isolation and immobilization stress as a confounder. Lack of EEG monitoring may allow microsleep and therefore have bias on the deprivation

Disc over water (DOW)	The apparatus is comprised of two rectangular clear plastic chambers placed side by side. A single plastic disc (40 cm diameter) serving as the rat carrier platform was built into the lower quarter of the two chambers. Beneath the disc, extending to the chamber walls was a rectangular tray filled with water to a depth of 5 cm. An electric motor was set to run the rats carrying disc at a speed of 3.3 rpm whenever sleep was detected by the EEG recorder on the experimental subject; control may sleep while deprived subject is spontaneously awakened. If not using EEG monitoring, motor may be set to run at a continuous mode	Home caged controls and yoked controls	May be used to cause acute and/or chronic sleep deprivation. Depending EEG programming may be used to deprive both PSD and TSD. Eliminates immobilization as a stress confounder. Low stressful environment	Isolation stress may appear as a confounder. Lack of EEG monitoring may induce stress caused by nonstop physical activity

Disc over water: DOW, electroencephalogram: EEG, electromyogram: EMG, paradoxical sleep deprivation: PSD, and total sleep deprivation: TSD.

**Table 3:** Oxidative stress in brain regions during paradoxical sleep deprivation in mice.

Author	Sleep deprivation protocol	Method	Animal specie	Deprivation time	Brain regions with oxidative changes	GSH	GSSG	GSH/GSSG ratio	GPx	CAT	SOD	Nitrites	NO	Lipid oxidation
Lima et al. (2014) [27]	PSD	MSP	Swiss albino male mice	48 hrs	Striatum	No	No	No	No	No	No	Yes —	No	Yes ↑
				72 hrs	Striatum	No	No	No	No	No	No	Yes ↓	No	Yes ↑
				48 & 72 hrs	Hippocampus & prefrontal cortex	No	No	No	No	No	No	Yes ↓	No	Yes ↑

Kumar and Singh (2009) [40]	PSD	GOW	Laca mice	72 hrs	Whole brain	Yes ↓	No	No	No	Yes ↓	No	Yes ↑	No	Yes ↑

Kumar and Garg (2009) [41]	PSD	GOW	Laca mice	72 hrs	Whole brain	Yes ↓	No	No	No	Yes ↓	No	Yes ↑	No	Yes ↑

Singh et al. (2008) [42]	PSD	GOW	Male Laca mice	72 hrs	Whole brain	Yes ↓	No	No	No	Yes ↓	No	Yes ↑	No	Yes ↑

Singh and Kumar (2008) [43]	PSD	GOW	Male Laca mice	72 hrs	Whole brain	Yes ↓	No	No	No	Yes ↓	No	Yes ↑	No	Yes ↑

Kumar and Singh (2008) [45]	PSD	GOW	Male Laca mice	72 hrs	Whole brain	Yes ↓	No	No	No	Yes ↓	No	Yes ↑	No	Yes ↑

Kumar and Garg (2008) [46]	PSD	GOW	Male Laca mice	72 hrs	Whole brain	Yes ↓	No	No	No	Yes ↓	No	Yes ↑	No	Yes ↑

Garg and Kumar (2008) [47]	PSD	GOW	Male Laca mice	72 hrs	Whole brain	Yes ↓	No	No	No	Yes ↓	No	Yes ↑	No	Yes ↑

Silva et al. (2007) [49]	PSD	MSP	Swiss EPM-M1 male mice	24 hrs	Hippocampus	No	No	No	No	No	No	No	No	Yes —

Kumar and Singh (2007) [50]	PSD	GOW	Male Laca mice	72 hrs	Whole brain	Yes ↓	No	No	No	Yes ↓	No	Yes ↑	No	Yes ↑

Kumar and Kalonia (2007) [51]	PSD	GOW	Male Laca mice	48 hrs	Whole brain	Yes ↓	No	No	No	Yes ↓	No	Yes ↑	No	Yes ↑

Silva et al. (2004) [52]	PSD	MSP with MLP	Swiss EPM-M1 male mice	72 hrs	Hippocampus	Yes ↓	No	Yes ↓	No	No	No	No	No	Yes ↑

Catalase: CAT, glutathione: GSH, oxidized glutathione: GSSG, glutathione peroxidase: GPx, grid over water: GOW, hours: hrs, multiple large platforms: MLP, multiple small platforms: MSP, nitric oxide: NO, paradoxical sleep deprivation: PSD, superoxide dismutase: SOD, ↓: significantly reduced, ↑: significantly increased, and —: not significantly increased or decreased.

**Table 4:** Oxidative stress in brain regions during paradoxical sleep deprivation in rats.

Author	Sleep deprivation protocol	Method	Animal specie	Deprivation time	Brain regions with oxidative changes	GSH	GSSG	GSH/GSSG ratio	GPx	Catalase	SOD	Nitrites	NO	Lipid oxidation
Zhang et al. (2013) [28]	PSD	MSP with MLP	Male Sprague Dawley rats	24 & 72 hrs	Cerebral cortex & hippocampus	No	No	No	No	No	Yes ↓↓	No	Yes ↑↑	Yes ↑↑

Hirotsu et al. (2013) [30]	PSD	MSP	Male Wistar Hanover rats	24 hrs	Frontal cortex	No	No	No	No	Yes —	Yes —	No	Yes —	No

Alzoubi et al. (2013) [31]	PSD	MSP with MLP	Male Wistar rats	8 hrs/day for 6 weeks	Hippocampus	Yes —	Yes ↑	Yes ↓	Yes ↓	Yes ↓	Yes ↓	No	No	No

Alzoubi et al. (2013) [32]	PSD	MSP with MLP	Male Wistar rats	8 hrs/day for 6 weeks	Hippocampus	Yes —	Yes ↑	Yes ↓	Yes ↓	Yes ↓	Yes ↓	No	No	Yes —

Alzoubi et al. (2012) [33]	PSD	MSP with MLP	Male Wistar rats	8 hrs/day for 6 weeks	Hippocampus	Yes —	Yes ↑	Yes ↓	Yes ↓	Yes ↓	Yes ↓	No	No	No

Vollert et al. (2011) [34]	PSD	MSP with MLP	Male Wistar rats	24 hrs	Cortex, amygdala, & hippocampus	No	No	No	No	No	No	No	No	Yes ↑

Süer et al. (2011) [35]	PSD	MSP with MLP	Male Wistar rats	18 hrs/day for 21 days	Whole brain & hippocampus	No	No	No	Yes ↓	No	Yes ↓	No	No	Yes ↑

Khadrawy et al. (2011) [37]	PSD	MSP with MLP	Male Wistar rats	72 hrs	Cortex Hippocampus	Yes ↓ Yes ↓	No No	No No	No No	Yes — Yes —	No No	No No	Yes — Yes ↑	Yes ↑ Yes ↑

Singh and Kumar (2008) [44]	PSD	CP	Male Wistar rats	96 hrs	Hippocampus, thalamus, & hypothalamus	Yes ↓	No	No	Yes —	No	Yes ↓	No	No	Yes ↑
					Cerebral cortex, brain stem	Yes —	No	No	Yes —	No	Yes ↑	No	No	Yes ↓
					Striatum	Yes —	No	No	Yes —	No	Yes —	No	No	Yes —
					Whole brain	Yes ↓	No	No	Yes —	No	Yes —	No	No	Yes —

Das et al. (2008) [48]	PSD	CP	Male Wistar rats	96 hrs	Whole brain	No	No	No	No	No	No	No	No	Yes ↓

D'Almeida et al. (2000) [56]	PSD	CP	Male Wistar rats	96 hrs	Hypothalamus	Yes ↓	No	No	No	No	No	No	No	No

D'Almeida et al. (1998) [57]	PSD	CP	Male Wistar rats	96 hrs	Hypothalamus	Yes ↓	No	No	No	No	No	No	No	No
					Cortex, hippocampus, thalamus, pons, & cerebellum	Yes —	No	No	No	No	No	No	No	No

D'Almeida et al. (1997) [58]	PSD	MSP with MLP	Male Wistar rats	96 hrs	Frontal cortex Whole brain	Yes ↓ No	No No	No No	No Yes —	No Yes —	No Yes —	No No	No No	No Yes —

Catalase: CAT, classical platform: CP, glutathione: GSH, oxidized glutathione: GSSG, glutathione peroxidase: GPx, hours: hrs, multiple large platforms: MLP, multiple small platforms: MSP, nitric oxide: NO, paradoxical sleep deprivation: PSD, superoxide dismutase: SOD, ↓: significantly reduced, ↑: significantly increased, —: not significantly increased or decreased, and ↑↑: significantly increased compared with a control and between sleep deprivation times.

**Table 5:** Oxidative stress in brain regions during total sleep deprivation in rats.

Author	Sleep deprivation protocol	Method	Animal specie	Deprivation time	Brain regions with oxidative changes	GSH	GSSG	GSH/GSSG ratio	GPx	Catalase	SOD	Nitrites	NO	Lipid oxidation
Melgarejo-Gutiérrez et al. (2013) [29]	TSD	Handling with EEG monitoring	Male Wistar rats	24 hrs	Hippocampus	No	No	No	No	No	No	No	No	Yes ↓

Hirotsu et al. (2013) [30]	TSD	Handling	Male Wistar Hanover rats	6 hrs	Frontal cortex	No	No	No	No	Yes —	Yes —	No	Yes —	No

Ramanathan and Siegel (2011) [36]	TSD	Handling	Male Sprague Dawley rats	6 hrs	Hippocampus	Yes —	No	No	No	No	Yes —	No	Yes ↑	Yes ↓
					Cerebellum, brainstem, & neocortex	Yes ↑	No	No	No	No	Yes —	No	Yes —	Yes —

Ramanathan et al. (2010) [38]	TSD	Handling	Male Sprague Dawley rats	6 hrs	Cortex, brainstem, and basal forebrain	Yes ↑	No	No	Yes —	No	Yes —	No	No	No
					Cerebellum & hippocampus	Yes —	No	No	Yes ↑	No	Yes —	No	No	No

Kalinchuk et al. (2010) [39]	TSD	Handling with EEG monitoring	Male Wistar rats	6 hrs	Basal forebrain	No	No	No	No	No	No	No	Yes ↑	No

Gopalakrishnan et al. (2004) [53]	TSD	Handling and DOW, both with EEG monitoring	Male Wistar Kyoto rats	ST: 8 hrs LT: 3–14 days	Cerebral cortex	No	No	No	No	No	Yes —	No	No	Yes —

Hsu et al. (2003) [54]	TSD	DOW	Sprague Dawley rats	5 days	Hippocampus	No	No	No	No	No	No	No	Yes ↓	No

Ramanathan et al. (2002) [55]	TSD	DOW with EEG monitoring	Male Sprague Dawley rats	5–11 days	Hippocampus	No	No	No	Yes —	No	Yes ↓	No	No	No
					Cortex, cerebellum hypothalamus, & brainstem	No	No	No	Yes —	No	Yes —	No	No	No

Catalase: CAT, disc over water: DOW, electroencephalogram: EEG, glutathione: GSH, oxidized glutathione: GSSG, glutathione peroxidase: GPx, grid over water: GOW, hours: hrs, long term: LT, nitric oxide: NO, short term: ST, superoxide dismutase: SOD, total sleep deprivation: TSD, ↓: significantly reduced, ↑: significantly increased, and —: not significantly increased or decreased.

**Table 6:** Oxidative stress in nonbrain regions during sleep deprivation.

Author	Sleep deprivation protocol	Method	Animal specie	Deprivation time	Body regions with oxidative changes	GSH	GSSG	GSH/GSSG ratio	GPx	Catalase	SOD	Nitrites	NO	Lipid oxidation
Lungato et al. (2013) [59]	PSD	MSP	Male Swiss mice	72 hrs	Spleen	No	No	No	No	Yes ↓	Yes ↑	No	No	Yes —

Egydio et al. (2012) [60]	PSD	MSP	Male hairless mice	72 hrs	Skin	No	No	No	No	No	No	No	Yes —	No

Chang et al. (2008) [61]	TSD	DOW with EEG monitoring	Male Wistar rats	5 days	Liver	No	No	No	No	No	No	No	No	Yes ↑

Chang et al. (2006) [62]	TSD	DOW	Male Wistar rats	5 days	Nodose ganglion	No	No	No	No	No	No	No	Yes ↓	No

Everson et al. (2005) [63]	TSD	DOW with EEG monitoring	Male Sprague Dawley rats	5 or 10 days	Liver Heart Lung	Yes ↓ Yes — Yes —	No No No	Yes — Yes — Yes —	Yes — Yes ↑↑ Yes —	Yes ↓↓ Yes — Yes —	No No No	No No No	No No No	No No No

Gopalakrishnan et al. (2004) [53]	TSD	DOW with EEG monitoring	Male Wistar Kyoto rats	3–14 days	Liver Skeletal muscle	No No	No No	No No	No No	No No	Yes — Yes —	No No	No No	Yes — Yes —

de Oliveira et al. (2002) [64]	PSD	MSP	Male Wistar rats	96 hrs	Plasma	No	No	No	No	No	No	No	No	Yes ↓

Catalase: CAT, disc over water: DOW, electroencephalogram: EEG, glutathione: GSH, oxidized glutathione: GSSG, glutathione peroxidase: GPx, grid over water: GOW, hours: hrs, multiple small platforms: MSP, nitric oxide: NO, paradoxical sleep deprivation: PSD, superoxide dismutase: SOD, total sleep deprivation: TSD, ↓: significantly reduced, ↑: significantly increased, —: not significantly increased or decreased, and ↑↑: significantly increased compared with a control and between sleep deprivation times.
